# Evaluating the Association between p53 Codon 72 Arg>Pro Polymorphism and Risk of Ovary Cancer: A Meta-Analysis

**DOI:** 10.1371/journal.pone.0094874

**Published:** 2014-04-18

**Authors:** Mohammed A. A. Alqumber, Naseem Akhter, Shafiul Haque, Aditya K. Panda, Raju K. Mandal

**Affiliations:** 1 Department of Laboratory Medicine, Faculty of Applied Medical Sciences, Albaha University, Albaha, Saudi Arabia; 2 Department of Biosciences, Jamia Millia Islamia (A Central University), New Delhi, India; 3 Department of Infectious Disease Biology, Institute of Life Sciences, Bhubaneswar, Odisha, India; 4 Department of Urology, Sanjay Gandhi Post Graduate Institute of Medical Sciences, Lucknow, Uttar Pradesh, India; University of Saarland Medical School, Germany

## Abstract

**Aim:**

Allelic polymorphism in codon 72 of the p53 tumor suppressor gene causes imbalance of p53 protein expression. Earlier studies have shown association between allelic polymorphism in codon 72 of the p53 gene with risk of ovary cancer (OC); however the results are inconclusive and conflicting. Therefore, we performed this meta-analysis to investigate the relation between p53 codon 72 Arg>Pro polymorphism and overall OC susceptibility.

**Methods:**

We searched all eligible published studies based on the association between codon 72 of the p53 Arg>Pro polymorphism and risk of OC. Data were pooled together from individual studies and meta-analysis was performed. Pooled odds ratios (ORs) and 95% CI were calculated for allele contrast, homozygous, heterozygous, dominant and recessive genetic models.

**Results:**

A total of twelve studies comprising of 993 OC cases and 1264 healthy controls were included in this meta-analysis. Overall, no significant association was detected for Pro allele carrier (Pro vs. Arg: p = 0.916; OR = 0.980, 95% CI = 0.677 to 1.419), homozygous (Pro/Pro vs. Arg/Arg: p = 0.419; OR = 0.731, 95% CI = 0.341 to 1.564), heterozygous (Arg/Pro vs. Arg/Arg: p = 0.248; OR = 1.237, 95% CI = 0.862 to 1.773), dominant (Pro/Pro+Arg/Pro vsArg/Arg: p = 0.699; OR = 1.089, 95% CI = 0.706 to 1.681), and recessive (Pro/Pro vs Arg/Arg+Arg/Pro: p = 0.329; OR = 0.754, 95% CI = 0.428 to 1.329) genetic models, respectively. Also, in the stratified analysis by ethnicity, no significant association of this polymorphism with risk of OC was found in the Caucasian population.

**Conclusions:**

This meta-analysis suggested that codon 72 of the p53 Arg>Pro polymorphism may not significantly contribute in ovary cancer susceptibility. However, future large studies with gene-gene and gene-environment interactions are needed to validate these findings.

## Introduction

Ovary cancer (OC) is the most common carcinoma among females with poor prognosis. It is the sixth leading cause of death among gynecological malignancies in females worldwide [1], [2]. The etiology of OC is still unclear and epidemiological studies have suggested that susceptibility to OC of an individual is influenced by several genetic factors [3]. However, there is no thorough screening technique for this malignancy, indicating that the identification of a gene related to the risk of OC may improve the early diagnosis and prevention of this deadly disease.

The p53 tumor suppressor gene (TP53 at 17p13), recognized as “the guardian of the genome”, plays a significant role in the cell cycle arrest, senescence, DNA damage repair, regulates the cell cycle and requires loss of function mutations for tumor formation [4].

The p53 protein functions by interfering with central regulators of hypoxia which mediate angiogenesis, and eventually inhibit production of pro-angiogenic factors and endogenous angiogenesis inhibitors [5], [6], [7]. The ability of p53 to eliminate excess, damaged or infected cells by apoptosis is essential for the proper regulation of cell proliferation in multi-cellular organisms. Differential expression of p53 in various cancers and association of serum p53 levels with malignant tumors highlights the significance role of p53 in malignancy [8], [9]. The major modes of TP53 inactivation are single-base substitutions and loss of alleles, with inactivation by viral or cellular proteins [10].

Several polymorphisms have been detected in both coding and non-coding region of this gene [11]. An important single nucleotide germ line polymorphism in the proline- rich domain of exon 4 of p53 gene induces an arginine to proline residue change at amino acid position 72 [12]. The two polymorphic forms (Pro72 and Arg72) of p53 gene have different primary structures, electrophoretic migration and functional properties [13]. The arginine (Arg72) allele increases the ability of p53 to locate to mitochondria and induce cellular death, whereas proline allele (Pro72) impart a lower apoptotic potential and an increased cellular arrest in G1 phase of the cell cycle [14].

Considering the functional significance of p53 gene in carcinogenesis, it is speculated that codon 72 Arg>Pro polymorphism may be a potential susceptibility factor for OC. Lately, several epidemiological case-control studies have evaluated the association between p53codon 72 Arg>Pro polymorphisms and OC risk [15]–[26]. Despite several studies globally, the putative association between p53 codon 72 Arg>Pro genetic polymorphism and OC risk remains uncertain and lacks consensus. Therefore, to derive a more precise conclusion of the possible association between p53 codon 72 Arg>Pro polymorphism and OC risk, a meta-analysis was performed based on eligible published studies.

## Materials and Methods

### Publication search strategy

We carried out a PubMed (Medline), EMBASE and Google Scholar web database search covering all research articles published with a combination of the following key words: ‘p53 gene (polymorphism OR mutation OR variant) AND ovarian carcinoma or ovary cancer, tumor susceptibility (last updated on November 2013). All the searched studies were retrieved and their reference lists were checked as well for other relevant studies. When, more than one of the same population was included in several publications, only the most recent or complete study was included in this meta-analysis. Since, this is a meta-analysis of published articles based on the association of p53 codon 72 Arg>Pro polymorphism and OC risk, so ethical approval was not required for this study.

### Inclusion and exclusion criteria

In order to minimize heterogeneity and ease the appropriate interpretation of this study, published articles included in the current meta-analysis had to meet all the following inclusion criteria: a) studies should have a cross-sectional, case-control or cohort design, b) must evaluated the association between p53 codon 72 Arg>Pro polymorphism and OC risk, c) recruited pathologically or histologically confirmed OC patients and healthy controls, d) have available genotype frequency in case and control, e) and published in English language. Also, when the case-control study was included by more than one research article using the same case series, we selected the study that included the largest number of individuals. On the other side, the major reasons for study exclusion were, overlapping of the data, case-only studies, review articles, and genotype frequencies or numbers are not reported. The study selection procedure has been shown in the form of flow-diagram as Figure S1 (PRISMA Flow Diagram).

### Data extraction and quality assessment

For each retrieved research publication, the methodological quality assessment and data extraction were independently abstracted in duplicate using a standard protocol by two independent investigators. Data-collection form was used to guarantee the accuracy of the collected data by stringently following the inclusion-exclusion criteria mentioned above. The main characteristics abstracted from the retrieved studies included the name of the first author, publication year, the country of origin, the number of cases and controls, study type, and genotype frequencies. Cases associated with disagreement on any item of the data from the collected research studies were fully debated with investigators to achieve a final consensus.

### Statistical analysis

In order to examine the relationship between p53 codon 72 Arg>Pro polymorphism and OC risk, pooled ORs and their corresponding 95% CIs were estimated. Heterogeneity assumption was examined by the chi-square-based Q-test [27]. Heterogeneity was considered significant when p-value<0.05. The data from single comparison was pooled using fixed effects model [28] when no heterogeneity presented. Otherwise, the random-effects model [29] was used for pooling purpose. Additionally, I^2^ statistics was employed to quantify inter-study variability and larger values suggested an increasing degree of heterogeneity [30]. Hardy-Weinberg equilibrium (HWE) in the controls was calculated via chi-square test. Funnel plot asymmetry was estimated by Egger's linear regression test which is a type of linear regression methodology to measure the funnel plot asymmetry on the natural logarithm scale of the OR. The significance of the intercept was determined by the t-test considering p-value<0.05 as representation of statistically significant publication bias [31]. Also, the subgroup analysis was carried out by the ethnicity, and the ethnicity was defined mainly as Caucasians. A comparative examination of ‘meta-analysis’ softwares was performed by using url address http://www.meta-analysis.com/pages/comparisons.html. The Comprehensive Meta-Analysis (CMA) Version 2 software program (Biostat, USA) was chosen and utilized to perform all statistical analysis involved in this study.

## Results

### Characteristics of included studies

According to our selection (inclusion-exclusion) criteria, a total of twelve research articles were finally included through literature search from the PubMed (Medline), EMBASE and Google Scholar web databases in this meta-analysis. All retrieved research publications were examined carefully by reading the titles and abstracts, and the full texts for the potentially relevant research articles were further checked for their aptness for the current meta-analysis. Studies either showing p53 codon 72 Arg>Pro polymorphism to predict survival in OC patients or considering p53 variants as an indicators for response to therapy were excluded straightaway. Similarly, studies investigating the levels of p53 mRNA or protein expression or relevant review articles were also excluded. In the present meta-analysis, only case-control or cohort design studies having frequency of all three genotypes were included. Besides the database search, the reference lists present in the retrieved articles were also checked for other potential research publications (Table 1). Distribution of genotypes, minor allele frequency (MAF) and HWE in the controls and cases have been presented in Table 2.

**Table 1:** Characteristics of studies included in this meta-analysis.

First Authors and year	Country of origin	Study Design	Cases	Controls	Source of genotyping
Dholariya et al. 2013 [15]	India	HB	100	100	Blood
Malisic et al. 2013 [16]	Serbia	HB	47	70	Tissue
Matei et al. 2012 [17]	Romania	HB	21	21	Blood
Ueda et al. 2006 [18]	Japan	HB	68	95	Blood
Morari et al. 2006 [19]	Brazil	HB	69	222	Blood
Santos et al. 2006 [20]	Portugal	HB	99	188	Blood
Agorastos et al. 2004 [21]	Greece	HB	51	30	Cytobrush
Pegoraro et al. 2003 [22]	South Africa	HB	85	340	Blood
Hogdall et al. 2002 [23]	Denmark	HB	211	83	Blood
Li et al. 2002 [24]	China	HB	39	50	Tissue
Buller et al. 1997 [25]	America	HB	190	52	Blood
Peller et al. 1999 [26]	Israel	HB	13	13	Blood

**Table 2:** Distribution of p53 polymorphism of twelve studies included in the meta-analysis.

Authors and year	Control				Case				HWE
	Genotype			Minor allele	Genotype			Minor allele	
	Arg/Arg	Arg/Pro	Pro/Pro	MAF	Arg/Arg	Arg/Pro	Pro/Pro	MAF	HWEF
Dholariya et al. 2013	62	32	6	0.22	33	50	17	0.42	0.49
Malisic et al. 2013	45	22	3	0.2	22	22	3	0.29	0.88
Matei et al. 2012	7	7	7	0.5	9	6	6	0.42	0.12
Ueda et al. 2006	34	54	7	0.35	21	41	6	0.38	0.02
Morari et al. 2006	117	91	14	0.26	23	46	0	0.33	0.51
Santos et al. 2006	117	58	13	0.22	49	40	10	0.30	0.12
Agorastos et al. 2004	6	19	25	0.69	26	22	3	0.27	0.42
Pegoraro et al. 2003	32	147	161	0.68	14	41	30	0.59	0.85
Hogdall et al. 2002	48	27	8	0.25	118	73	20	0.26	0.16
Li et al. 2002	10	26	14	0.54	14	20	5	0.38	0.74
Buller et al. 1997	30	18	4	0.25	98	79	13	0.27	0.57
Peller et al. 1999	8	5	0	0.19	7	6	0	0.23	0.39

### Publication bias

Begg's funnel plot and Egger's test were carried out to evaluate the publication bias among the selected studies for the meta-analysis. The appearance of the shape of funnel plots was seemed to be symmetrical in all the genetic models. The Egger's test was performed to provide the statistical evidence of funnel plot. The results showed lack of publication bias among all comparison models (Table 3).

**Table 3:** Statistics to test publication bias and heterogeneity in the present meta-analysis.

Comparisons	Egger's regression analysis			Heterogeneity analysis			Model used for the meta-analysis
	Intercept	95% Confidence Interval	p-value	Q-value	P_heterogeneity_	I^2^ (%)	
Pro vs. Arg	−1.56	−7.40 to 4.26	0.56	70.75	<0.0001	84.45	Random
Pro/Pro vs. Arg/Arg	−1.61	−6.48 to 3.26	0.47	45.35	<0.0001	77.95	Random
Arg/Pro vs. Arg/Arg	−2.72	−5.95 to 0.51	0.09	30.12	0.002	63.48	Random
Pro/Pro+Arg/Pro vs. Arg/Arg	−3.38	−7.58 to 0.82	0.10	48.78	<0.0001	77.45	Random
Pro/Pro vs. Arg/Arg+Arg/Pro	−0.49	−3.70 to 2.72	0.73	31.08	0.001	67.83	Random

### Evaluation of heterogeneity

In order to analyze heterogeneity among the selected studies, Q-test and I^2^ statistics were employed and heterogeneity was noticed in all the five genetic models. Therefore, random effects model was applied to synthesize the data (Table 3).

### Association of p53 codon 72 Arg>Pro polymorphism and OC susceptibility

**Figure 1:**
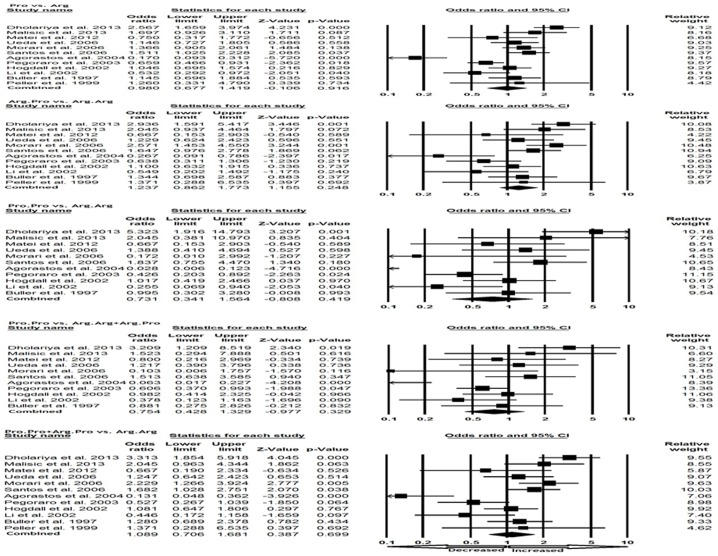
Forest plot of OR with 95% CI of ovary cancer associated with the p53 codon 72 Arg>Pro gene polymorphism. Black square represent the value of OR and the size of the square indicates the inverse proportion relative to its variance. Horizontal line is the 95% CI of OR. The studies are listed by year of publication.

We pooled all the twelve studies together and it resulted into 1264 controls and 993 OC cases, to review the overall association between p53 codon 72 Arg>Pro polymorphism and OC risk. Overall pooled analysis did not suggest any correlation between p53 codon 72 Arg>Pro polymorphism and OC risk in all the five genetic comparison models, i.e., allele (Pro vs. Arg: p = 0.916; OR = 0.980, 95% CI = 0.677 to 1.419), homozygous (Pro.Pro vs. Arg.Arg: p = 0.419; OR = 0.731, 95% CI = 0.341 to 1.564), heterozygous (Arg.Pro vs. Arg.Arg: p = 0.248; OR = 1.237, 95% CI = 0.862 to 1.773), recessive (Pro.Pro vs. Arg.Arg+Arg.Pro: p = 0.329; OR = 0.754, 95% CI = 0.428 to 1.329) and dominant model (Pro.Pro+Arg.Pro vs. Arg.Arg: p = 0.699; OR = 1.089, 95% CI = 0.706 to 1.681) (Figure 1).

### Subgroup analysis of racial descent

**Figure 2:**
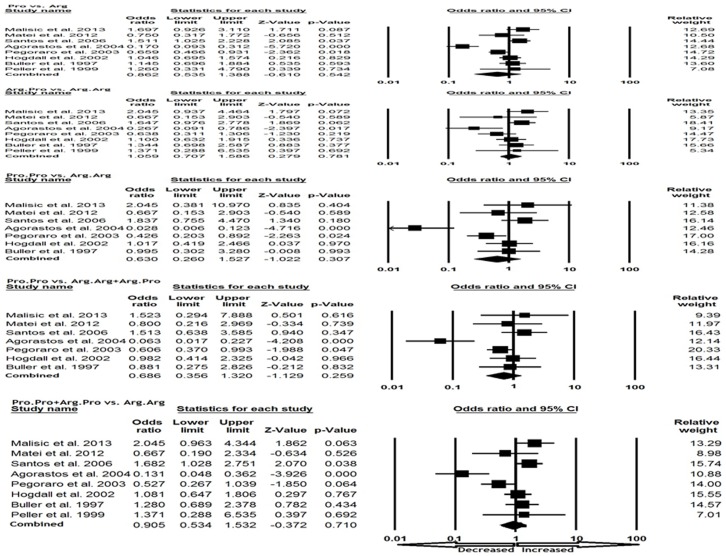
Forest plot of OR with 95% CI of ovary cancer associated with the p53 codon 72 Arg>Pro gene polymorphism according to the ethnicity group by the random effect model. Black square represent the value of OR and the size of the square indicates the inverse proportion relative to its variance. Horizontal line is the 95% CI of OR. The studies are listed by year of publication.

We have analyzed only Caucasian population by study design and participants. This meta-analysis included eight studies (717 cases and 797 controls), Heterogeneity was observed in all genetic models; thus, random effect model was applied to analyze the data. In subgroup analysis publication bias did not exist (Table 4). We did not observe any association of p53 codon 72 Arg>Pro polymorphism with OC risk in Caucasian population in all genetic comparison models, i.e., allele (Pro vs. Arg: p = 0.542; OR = 0.862, 95% CI = 0.535 to 1.388), homozygous (Pro.Pro vs. Arg.Arg: p = 0.307; OR = 0.630, 95% CI = 0.260 to 1.527), heterozygous (Arg.Pro vs. Arg.Arg: p = 0.781; OR = 1.059, 95% CI = 0.707 to 1.586), recessive (Pro.Pro vs. Arg.Arg+Arg.Pro: p = 0.259; OR = 0.686, 95% CI = 0.356 to 1.320) and dominant model (Pro.Pro+Arg.Pro vs. Arg.Arg: p = 0.710; OR = 0.905, 95% CI = 0.534 to 1.532) (Figure 2)

**Table 4:** Statistics to test publication bias and heterogeneity in the present meta-analysis.

Comparisons	Egger's regression analysis			Heterogeneity analysis			Model used for the meta-analysis
	Intercept	95% Confidence Interval	p-value	Q value	P_heterogeneity_	I^2^ (%)	
Pro vs. Arg	−0.65	−7.59 to 6.28	0.82	44.95	<0.0001	84.43	Random
Pro/Pro vs. Arg/Arg	−1.29	−9.14 to 6.54	0.68	26.87	<0.0001	77.67	Random
Arg/Pro vs. Arg/Arg	−1.69	−5.29 to 1.91	0.29	14.34	0.04	51.19	Random
Pro/Pro+Arg/Pro vs. Arg/Arg	−2.34	−7.37 to 2.68	0.29	27.67	<0.0001	74.70	Random
Pro/Pro vs. Arg/Arg+Arg/Pro	−0.17	−4.97 to 4.63	0.93	18.46	0.005	67.51	Random

## Discussion

The p53 tumor suppressor gene, mutated in many human cancer types, suggests its key role in the host's defense against malignancy [6]. The cellular level of the p53 protein is regulated in a complex manner by a negative feedback loop involving ubiquitin medicated degradation [32]. Based upon the nature of the genetic insult, wild-type p53 induces either growth arrest or apoptosis. Any alterations within p53 gene restrict these activities and allow the continuous proliferation of cells, ultimately resulting in the progression to malignancy [33].

Despite remarkable progress in mechanistic understanding of p53 structure and function, the contribution of specific p53 gene polymorphisms to OC risk remains equivocal and proven to be extremely complex biomarkers. Interest in the genetic susceptibility to OC has led to an emerging trend to the study of polymorphisms of genes involved in OC risk. Due to the different roles of p53 gene in human genome, it has been hypothesized that codon 72 Arg>Pro polymorphism is associated with risk of OC. As a result, a large number of studies have been performed to evaluate the association between p53 codon 72 Arg>Pro polymorphism and risk of OC, but the results from different published studies lacks consensus. Inconsistency in results from these studies can be attributed to low statistical power to evaluate the overall effect of the p53 codon 72 Arg>Pro polymorphism with OC risk. The answer to this limitation is a meta-analysis, a powerful tool for investigating the risk factors associated with genetic diseases, which employs quantitative technique to pool the data from individual studies where individual sample sizes are small with lower statistical power, and provides reliable conclusion [34]. Hence, we have done the present meta-analysis from twelve eligible published case-control studies to evaluate the said relation of p53 codon 72 Arg>Pro polymorphism and risk of OC. This study might help to explore a more robust estimate about the role of this polymorphism with OC risk, as combining data from many studies has the advantage of reduced random errors [35].

The overall pooled results of this meta-analysis revealed that p53 codon 72 Arg>Pro polymorphism did not influence an increased or decreased risk of OC in all the five genetic models as per the eligible studies when compared with wild type allele. Even in the stratified analysis by ethnicity, no statistically significant relationship between p53 codon 72 Arg>Pro genotype and OC risk was detected in Caucasian population. One possible explanation is that several other SNPs have been reported in the p53 gene and previous research showed that various other SNPs are related to the susceptibility to OC. It is possible that the analyzed variant does not act as primary susceptibility polymorphism and may be inhibiting p53 function by linking with other functional polymorphism alleles found in linkage disequilibrium (LD). Our results are in agreement with Shen et al. [36], whereas Zhang et al. 2008 has reported a decreased risk [37]. Moreover, pooled and meta-analyses for breast [38], lung [39] and endometrial [40] cancers do not support a significant role for this polymorphism in susceptibility. Susceptibility to OC is a multistep process in which environmental and genetic factors interact closely and a single genetic variant is usually insufficient to predict the risk of this deadly disease.

Heterogeneity between studies is very common in the genetic association studies of meta-analysis. In the present meta-analysis we found inter-study heterogeneity in overall analysis. There are several factors responsible for such heterogeneity, i.e., the genetic backgrounds for cases and controls, diverse genotype distribution of codon 72 Arg>Pro in different ethnic groups and uneven selection criteria for the cases and controls in different studies.

Despite the important findings from our current analysis, we still have to acknowledge some limitations of this study. First, we only included studies published in English language, abstracted and indexed by the selected electronic databases for the data analysis; it is possible that some pertinent reports published in other languages and indexed in other electronic databases may have missed. Second, the result of this meta-analysis was based on unadjusted ORs because not all eligible studies stated adjusted ORs. Third, the role of gene-environment interactions were not considered which may affect the risk of OC. Also, it is worthwhile to mention several strengths of our study. First, we have included significantly more number of cases and controls comparison to the previous meta-analysis study by using effective and efficient search strategy to increase the statistical power of the analysis. Second, the quality of the case-control studies included in the present pooling analysis was satisfactory and met with the pre-set inclusion criteria.

## Conclusion

In conclusion, a meta-analysis is a rational approach of data-analysis which pools both statistically significant and non-significant findings from individual studies to improve the statistical performance by increasing the sample size. Our meta-analysis demonstrates that p53 codon 72 Arg>Pro polymorphism might not significantly modulate the OC risk. However, future well designed large studies, particularly stratified by gene-gene and gene-environment interactions might be necessary to clarify the possible role of the p53 codon 72 Arg>Pro polymorphism in the susceptibility to OC.

## Supporting Information

Figure S1 **PRISMA 2009 Flow Diagram.** Showing identification and selection of studies for the meta-analysis.
(TIF)

Checklist S1 **PRISMA 2009 Checklist.**
(DOC)

## References

[1] Clarke-Pearson DL (2009) Clinical practice. Screening for ovarian cancer. N Engl J Med 361: 170–177. 19587342 10.1056/NEJMcp0901926

[2] Jemal A, Bray F, Center MM, Ferlay J, Ward E, et al (2011) Global cancer statistics. CA Cancer J Clin 61 (2) 69–90. 21296855 10.3322/caac.20107

[3] Diaz-Padilla I, Amir E, Marsh S, Liu G, Mackay H (2012) Genetic polymorphisms as predictive and prognostic biomarkers in gynecological cancers: a systematic review. Gynecol Oncol 124 (2) 354–365. 22063461 10.1016/j.ygyno.2011.10.034

[4] Berchuck A, Kohler MF, Marks JR, Wiseman R, Boyd J, et al (1994) The p53 tumor suppressor gene frequently is altered in gynecologic cancers. Am J Obstet Gynecol 170 (1 Pt 1) 246–252. 8296829 10.1016/s0002-9378(94)70414-7

[5] Donehower LA, Harvey M, Slagle BL, McArthur MJ, Montgomery CA Jr, et al (1992) Mice deficient for p53 are developmentally normal but susceptible to spontaneous tumours. Nature 356 (6366) 215–221. 1552940 10.1038/356215a0

[6] Hollstein M, Sidransky D, Vogelstein B, Harris CC (1991) p53 mutations in human cancers. Science 253 (5015) 49–53. 1905840 10.1126/science.1905840

[7] Greenblatt MS, Bennett WP, Hollstein M, Harris CC (1994) Mutations in the p53 tumor suppressor gene: clues to cancer etiology and molecular pathogenesis. Cancer Res 54 (18) 4855–4878. 8069852

[8] Lane DP (1994) On the expression of the p53 protein in human cancer. Mol Biol Rep 19 (1) 23–29. 8170465 10.1007/BF00987319

[9] Wu M, Mao C, Chen Q, Cu XW, Zhang WS (2010) Serum p53 protein and anti-p53 antibodies are associated with increased cancer risk: a case-control study of 569 patients and 879 healthy controls. Mol Biol Rep 37 (1) 339–343. 19693693 10.1007/s11033-009-9744-7

[10] Tommasino M, Accardi R, Caldeira S, Dong W, Malanchi I, et al (2003) The role of TP53 in Cervical carcinogenesis. Hum Mutat 21 (3) 307–312. 12619117 10.1002/humu.10178

[11] Olivier M, Eeles R, Hollstein M, Khan MA, Harris CC, et al (2002) The IARC TP53 database: new online mutation analysis and recommendations to users. Hum Mutat 19 (6) 607–614. 12007217 10.1002/humu.10081

[12] Whibley C, Pharoah PD, Hollstein M (2009) p53 polymorphisms: cancer implications. Nat Rev Cancer 9 (2) 95–107. 19165225 10.1038/nrc2584

[13] Thomas M, Kalita A, Labrecque S, Pim D, Banks L, et al (1999) Two polymorphic variants of wild-type p53 differ biochemically and biologically. Mol Cell Biol 19 (2) 1092–1100. 9891044 10.1128/mcb.19.2.1092 PMC116039

[14] Pim D, Banks L (2004) p53 polymorphic variants at codon 72 exert different effects on cell cycle progression. Int J Cancer 108 (2) 196–199. 14639602 10.1002/ijc.11548

[15] Dholariya S, Zubari M, Ray PC, Gandhi G, Khurana N, et al (2013) TP53 Gene Polymorphism in Epithelial Ovarian Carcinoma Patients from North Indian Population and its Pro/Pro Variant is Potentially Contributing to Cancer Susceptibility. J Genet Syndr Gene Ther 4 (5) 1–6.

[16] Malisic EJ, Jankovic RN, Jakovljevic KV, Radulovic SS (2013) Association of TP53 codon 72 polymorphism with susceptibility to ovarian carcinomas in Serbian women. Eur J Obstet Gynecol Reprod Biol 166 (1) 90–93. 23092908 10.1016/j.ejogrb.2012.10.002

[17] Matei MC, Negură L, Liliac L, Negură A, Azoicăi D (2012) Validation of PCR-RFLP techniques for the evaluation of codon 72 of p53 and CYP1A1 gene's polymorphisms in relation with ovarian cancer in a Romanian population. Rom J Morphol Embryol 53 (1) 47–54. 22395499

[18] Ueda M, Terai Y, Kanda K, Kanemura M, Takehara M, et al (2006) Germline polymorphism of p53 codon 72 in gynecological cancer. Gynecol Oncol 100 (1) 173–178. 16168468 10.1016/j.ygyno.2005.08.015

[19] Morari EC, Lima AB, Bufalo NE, Leite JL, Granja F, et al (2006) Role of glutathione-S-transferase and codon 72 of P53 genotypes in epithelial ovarian cancer patients. J Cancer Res Clin Oncol 132: 521–528. 16788846 10.1007/s00432-006-0099-3 PMC12161103

[20] Santos AM, Sousa H, Pinto D, Portela C, Pereira D, et al (2006) Linking TP53 codon 72 and P21 nt590 genotypes to the development of cervical and ovarian cancer. Eur J Cancer 42 (7) 958–963. 16542834 10.1016/j.ejca.2006.01.015

[21] Agorastos T, Masouridou S, Lambropoulos AF, Chrisafi S, Miliaras D, et al (2004) P53 codon 72 polymorphism and correlation with ovarian and endometrial cancer in Greek women. Eur J Cancer Prev 13 (4) 277–280. 15554555 10.1097/01.cej.0000136717.95465.09

[22] Pegoraro RJ, Moodley M, Rom L, Chetty R, Moodley J (2003) P53 codon 72 polymorphism and BRCA 1 and 2 mutations in ovarian epithelial malignancies in black South Africans. Int J Gynecol Cancer 13 (4) 444–449. 12911720 10.1046/j.1525-1438.2003.13333.x

[23] Høgdall EV, Høgdall CK, Christensen L, Glud E, Blaakaer J, et al (2002) Distribution of p53 codon 72 polymorphisms in ovarian tumour patients and their prognostic significance in ovarian cancer patients. Anticancer Res 22 (3) 1859–1864. 12168882

[24] Li T, Lu ZM, Guo M, Wu QJ, Chen KN, et al (2002) p53 codon 72 polymorphism (C/G) and the risk of human papillomavirus-associated carcinomas in China. Cancer 95 (12) 2571–2576. 12467072 10.1002/cncr.11008

[25] Buller RE, Sood A, Fullenkamp C, Sorosky J, Powills K, et al (1997) The influence of the p53 codon 72 polymorphism on ovarian carcinogenesis and prognosis. Cancer Gene Ther 4 (4) 239–245. 9253509

[26] Peller S, Halperin R, Schneider D, Kopilova Y, Rotter V (1999) Polymorphisms of the p53 gene in women with ovarian or endometrial carcinoma. Oncol Rep 6 (1) 193–197. 9864427 10.3892/or.6.1.193

[27] Wu R, Li B (1999) A multiplicative-epistatic model for analyzing interspecific differences in outcrossing species. Biometrics 2: 355–365. 10.1111/j.0006-341x.1999.00355.x 11318188

[28] Mantel N, Haenszel W (1959) Statistical aspects of the analysis of data from retrospective studies of disease. J Natl Cancer Inst 4: 719–748. 13655060

[29] DerSimonian R, Laird N (1986) Meta-analysis in clinical trials. Control Clin Trials 7: 177–188. 3802833 10.1016/0197-2456(86)90046-2

[30] Higgins JP, Thompson SG, Deeks JJ, Altman DG (2003) Measuring inconsistency in meta-analyses. BMJ 7414: 557–560. 10.1136/bmj.327.7414.557 PMC192859 12958120

[31] Egger M, Davey Smith G, Schneider M, Minder C (1997) Bias in meta-analysis detected by a simple, graphical test. BMJ 7109: 629–634. 10.1136/bmj.315.7109.629 PMC2127453 9310563

[32] Vousden KH, Lu X (2002) Live or let die: the cell's response to p53. Nat Rev Cancer 2 (8) 594–604. 12154352 10.1038/nrc864

[33] Vogelstein B, Kinzler KW (1992) p53 function and dysfunction. Cell 70 (4) 523–526. 1505019 10.1016/0092-8674(92)90421-8

[34] Cohn LD, Becker BJ (2003) How meta-analysis increases statistical power. Psychol Methods 3: 243–253. 10.1037/1082-989X.8.3.243 14596489

[35] Bouillon R, Carmeliet G, Verlinden L, van Etten E, Verstuyf A, et al (2008) Vitamin D and Human Health: Lessons from Vitamin D Receptor Null Mice. Endocrine Reviews 29 (6) 726–776. 18694980 10.1210/er.2008-0004 PMC2583388

[36] Shen SQ, Jiang DK, Liu GY, Chen F, Yu L (2012) Meta-analysis shows significant association of the TP53 Arg72Pro with ovarian cancer risk. Mol Biol Rep 39 (4) 4683–4690. 21952824 10.1007/s11033-011-1260-x

[37] Zhang Z, Fu G, Wang M, Tong N, Wang S, et al (2008) P53 codon 72 polymorphism and ovarian cancer risk: a meta-analysis. JNMU 22 (5) 279–285.

[38] Schmidt MK, Reincke S, Broeks A, Braaf LM, Hogervorst FB, et al (2007) Breast Cancer Association Consortium. Do MDM2 SNP309 and TP53 R72P interact in breast cancer susceptibility? A large pooled series from the breast cancer association consortium. Cancer Res 67 (19) 9584–9590. 17909070 10.1158/0008-5472.CAN-07-0738

[39] Matakidou A, Eisen T, Houlston RS (2003) TP53 polymorphisms and lung cancer risk: a systematic review and meta-analysis. Mutagenesis 18 (4) 377–385. 12840112 10.1093/mutage/geg008

[40] Tang W, He X, Chan Y, Luo Y (2012) Lack of association between p53 codon 72 polymorphism and endometrial cancer: a meta-analysis. Cancer Epidemiol 36 (3) e153–7. 22277327 10.1016/j.canep.2011.12.010

