# Supplementary figures and images for: Evaluating the Association between p53 Codon 72 Arg>Pro Polymorphism and Risk of Ovary Cancer: A Meta-Analysis
Source: PLoS One. 2014 Apr 18;9(4):e94874. doi: 10.1371/journal.pone.0094874 (PMC3991634; doi:10.1371/journal.pone.0094874)

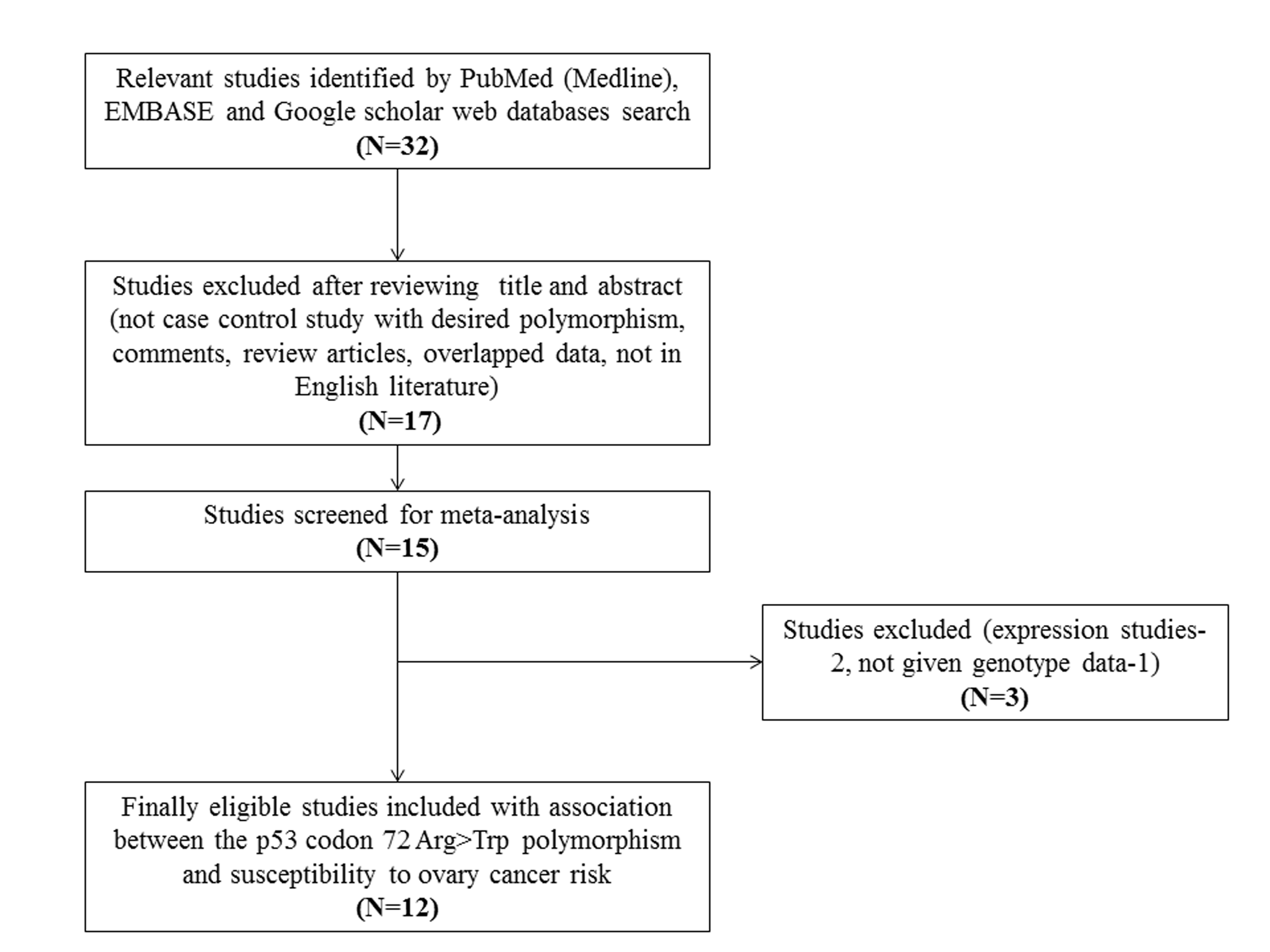

Supplement: Figure S1 — PRISMA 2009 Flow Diagram. Showing identification and selection of studies for the meta-analysis. (TIF) [file pone.0094874.s001.tif]
